# β-catenin ISGylation promotes lipid deposition and apoptosis in ethanol-stimulated liver injury models

**DOI:** 10.1080/13510002.2022.2109360

**Published:** 2022-10-19

**Authors:** Qi Ding, Guodong Zhang, Yang Wang, Lei Xu, Meifei Wu, Yiwen Zhou, Tao Xu, Xiaoming Meng, Cheng Huang, Lei Zhang

**Affiliations:** a Anhui No.2 Provincial People’s Hospital, Hefei, People’s Republic of China; b School of Pharmacy, Anhui Medical University, Hefei, People’s Republic of China; c Key Laboratory of major autoimmune disease, Anhui Province, School of Pharmacy, Anhui Medical University, Hefei, People’s Republic of China; d The Key Laboratory of Anti-inflammatory and Immune medicines, Ministry of Education, Anhui Medical University, Hefei, People’s Republic of China

**Keywords:** Alcoholic fatty liver disease, ISG15, HERC5, β-catenin ISGylation, ROS, Wnt signaling pathway, metabolism, apoptosis

## Abstract

**Background:**

The restoration of the Wnt/β-catenin pathway to alleviate alcoholic fatty liver disease (AFLD) progression is under study as a new strategy for alcoholic liver disease (ALD) treatment. Recent studies have indicated that interferon-stimulated gene 15 (ISG15) can covalently bind to β-catenin by HECT E3 ubiquitin ligase 5 (HERC5), leading to ISG degradation and downregulation of β-catenin levels. However, the relationship between β-catenin and the ISG15 system in AFLD remains unclear.

**Methods:**

Here, we explored the roles of the ISG15 system in β-catenin activation and in the pathogenesis of alcohol-induced liver injury and steatosis.

**Results:**

In this study, HERC5 silencing upregulated β-catenin protein expression and inhibited lipid metabolism disorders and cell apoptosis. Reduced β-catenin protein expression, increased lipid metabolism disorders, and cell apoptosis were detected in cells induced with HERC5 overexpression, which was reversible with the reactive oxygen species (ROS) inhibitor. All the above results were statistically analyzed. Thus, these observations demonstrate that β-catenin ISGylation is a prominent regulator of ALD pathology, which works by regulating ROS to induce lipid metabolism disorders and cell apoptosis.

**Conclusion:**

Our findings provided the mechanism involved in the β-catenin ISGylation, allowing for future studies on the prevention or amelioration of liver injury in ALD.

## Background

Excessive alcohol consumption is fatal to the liver, causing alcoholic liver disease (ALD) which is characterized by lipid metabolism disorders and liver injury [1,2]. Generally, ALD encompasses a wide spectrum of chronic liver disorders progressing from reversible steatosis to fibrosis, irreversible cirrhosis, and ultimately, hepatocellular cancer [3,4]. Despite advances in therapeutic strategies for ALD such as alcohol abstinence, corticosteroids, biologics, and liver transplantation, the effectiveness of these therapies for patients with end-stage ALD remains unknown [5]. The most common form of ALD is alcoholic fatty liver disease (AFLD), which is considered to be reversible [6]. A systematic study of the pathogenesis of AFLD can allow for the discovery of a more effective treatment to block or delay the progression of AFLD to advanced ALD.

Recent studies have highlighted that Wnt/β-catenin signaling is required for liver protection against oxidative stress-induced apoptosis. Furthermore, pharmacological restoration of Wnt/β-catenin signaling has been proven to be effective in attenuating ALD progression in a rat model, indicating that it plays a protective role in ALD [7,8]. The canonical Wnt/β-catenin signaling pathway has been evaluated for its role in hepatic development, regeneration, energy metabolism, and carcinogenesis [9]. β-catenin, the core protein of the canonical Wnt pathway, is a master regulator of the liver [10]. Conditional and liver-specific β-catenin knockout mice showed increased hepatic oxidative stress and blocked ethanol (EtOH) metabolism [11]. The activity of β-catenin is closely regulated by protein stability and transcriptional activity, with protein stability being the main regulator.

An important novel insight was obtained in recent research articles, showing that HECT E3 ubiquitin ligase 5 (HERC5)-mediated ISG15 conjugation is involved in the degradation of β-catenin [12,13]. Interferon-stimulated gene 15 (ISG15), a member of the ubiquitin-like (UBL) protein family, is known to play a critical role in the conjugation of target proteins. ISG15 is conjugated to numerous cellular proteins via a three-enzyme cascade system: Ubiquitin-activating enzyme E1 homolog (UBE1L) acts as an ISG15-activating E1 enzyme, UBCH8, a ubiquitin E2-conjugating enzyme, also functions as the ISG15-conjugating E2 enzyme, and some ISG15 E3 ligases, including HHARI, HERC5, and EFP, as E3 enzymes. To date, a number of studies have revealed that the ISG15 pathway is constitutively elevated in hepatocellular carcinoma [14]. Furthermore, it was also found that elevated ISG15 may contribute to the tumorigenesis and metastasis of malignant cells [15,16]. Notably, protein degradation of β-catenin by HERC5-mediated ISGylation has been observed in HeLa cells [13]. However, the relationship between β-catenin and the ISG15 system in AFLD remains unclear.

Therefore, an in-depth study on the regulatory mechanism of the ISGylation of β-catenin is crucial to understanding the pathogenesis of AFLD with abnormal Wnt signaling pathways. The current study was designed to test the effect of ISGylation of β-catenin on liver lipid metabolism and hepatocyte apoptosis as well as its mechanisms in the progression of AFLD.

## Materials and methods

### Murine model of alcohol-induced alcoholic fatty liver

The adult male C57BL/6J mice (18–22 g weight) were purchased from the Experimental Animal Center of Anhui Medical University. All animal procedures were reviewed and approved by the Institutional Animal Experimental Ethics Committee. Mice were randomly divided into control diet (CD)-fed and EtOH-fed groups. The CD-fed mice were fed with isocaloric maltose-dextrin (Sigma-Aldrich), and the EtOH-fed mice were fed ethanol (5% v/v) liquid diets (LD) for 10 days with a single binge ethanol administration (5 g/kg body weight, 20% ethanol) by gavage. The mice were anesthetized after the last gavage alcohol administration, and their liver tissues were harvested for further analysis. The liver tissues were either immediately frozen in liquid nitrogen, fixed in 10% neutral-buffered formalin for hematoxylin and eosin (H&E) staining and Oil red O staining, or embedded in frozen specimen medium (TissueTek OCT compound; Sakura Finetek, Torrance, CA, United States). All experiments were performed at Anhui Medical University according to the institutional ethical guidelines for laboratory animal care. Procedures involving animals and their care were conducted in conformance with NIH guidelines and approved by the Animal Care and Use Committee (number: LLSC20150348).

### Cell culture

The human liver L02 and mouse hepatocyte AML-12 cell lines were obtained from the Institute for Cell Bank of the Chinese Academy of Sciences (Shanghai, China). These were cultured in DMEM medium (Corning, Manassas, VA) at 37°C in a 5% CO_2_ incubator. The medium was supplemented with 10% fetal bovine serum, 100 U/mL penicillin, and 0.1 mg/mL streptomycin.

### Small interfering RNA and transfection

The L02 cells were cultured in 6-well plates and transfected with Lipofectamine^TM^ 2000 (Invitrogen, USA) according to the manufacturer’s instructions. Transfection efficiency was determined by qPCR analysis and western blotting. The oligonucleotides against the *HERC5* gene or scrambled sequences were designed and synthesized by Gene Pharma Company (Shanghai, China) and had the following sequences: *HERC5*-siRNA (human): 5-GGAAGGAAAUUUCCCUCAATT-3, 5-UUGAGGGAAAUUUCCUUCCTT-3; and negative control: 5-UUCUCCGAACGUGUCACGUTT-3, 5-ACGUGACACGUUCGGAGAATT-3’.

### Plasmid construction

The coding region of *HERC5* was amplified from human genomic DNA using the following primers: Forward: 5-TACAAGTCCGGACTCAGATCTATGGAGCGGAGGTCGCGGAGGAA-3; Reverse: 5-GTACCGTCGACTGCAGAATTCGTTGGACAAGCAAGCTGGTC -3. The amplified products were inserted into the pEGFP-C2 empty vector and verified with direct DNA sequencing. Cell transfection was performed with Lipofectamine^TM^ 2000 according to the manufacturer’s protocol. The efficiency of overexpression was determined by western blotting and qPCR analysis.

### RNA extraction and real-time quantitative PCR analysis

The total RNA was extracted from the cells or liver tissues using TRIzol (Invitrogen). These were then reverse transcribed to cDNA using the Transcriptor First Strand cDNA Synthesis Kit (TaKaRa, Shiga, Japan). The relative expressions of mRNA were determined by real-time PCR with SYBR-Green Master Mix (TaKaRa) according to the manufacturer’s protocol. The *GAPDH* gene was used as an internal control for normalization. The primers used are presented in supplementary table 1.

### Protein isolation and western blot analysis

The total protein was extracted from cells or liver tissues using RIPA lysis buffer (Beyotime, China). Each sample was isolated via electrophoresis through 8–12% sodium dodecyl sulfate-polyacrylamide gel electrophoresis (SDS-PAGE) and then transferred onto a polyvinylidene fluoride (PVDF) membrane (Millipore, Billerica, MA, USA). The membranes were incubated with the following primary antibodies at 1:300 dilutions: HERC5, ISG15, β-catenin, SREBP-1, PPAR-α, FOXO3a, BIM, Bax and Bcl-2 (Bioss, China). Caspase3 (Cell Signaling Technology, USA) and β-actin (Bioworld, USA) was diluted 1:1000. The protein bands were detected using an ECL-chemiluminescent kit (ECL-plus, Thermo Scientific).

### Histopathology

The liver tissues were fixed with 4% paraformaldehyde for 24 h and embedded in paraffin blocks or embedded in frozen specimen medium. H&E and Oil Red staining were performed according to the standard procedures. The pathological changes were assessed, and the samples were photographed under a microscope.

### Measurement of triglyceride levels

Cell homogenates and cell supernatants were collected to determine the levels of triglycerides (TGs) using the corresponding biochemical assay kits (Nanjing Jiancheng Bioengineering Institute, China).

### Flow cytometry analysis

Flow cytometric analysis was performed to evaluate the percentage of apoptotic cells and the level of *reactive oxygen species (ROS)*. The L02 cell lines were cultured in 6-well plates in DMEM containing 10% FBS. For apoptosis analysis, the density of cells used was 10^6^ cells/ml after addition of 400 ul Annexin V binding fluid. Cells were re-stained with 10ul Propidium iodide for 5 min and immediately measured by Flow cytometry. For ROS analysis, the cells were collected after transfection and ethanol stimulation and then processed with a reactive oxygen species (ROS) assay kit (Beyotime Biotechnology, China). The ROS in cells were detected by flow cytometry (Cytomics FC 500, Beckman Coulter, Germany) using the excitation wavelength of 488 nm.

### Computer-simulated docking

We downloaded the three-dimensional analytical structures of ISG15 and β-catenin from the Protein Data Bank (PDB) to study the initial interaction between ISG15 and β-catenin. AUTODOCK 4.0 was used on the Discovery Studio 2017R2 operating platform for docking research. In the experiment, molecular docking of receptor ISG15 and ligand β-catenin protein was carried out. After the docking was completed, only one construct was retained to be selected as the optimal protein docking construct.

### Statistical analysis

Data are presented as mean ± SD of all replicates and analyzed using SPSS16.0 software. Statistical analysis was performed using two-tailed unpaired t-test or one-way ANOVA. In all cases, *p *< 0.05 was considered statistically significant.

## Results

### ISG15 and HERC5 were markedly increased and β-catenin was decreased in the EtOH-fed mice

**Figure 1.**
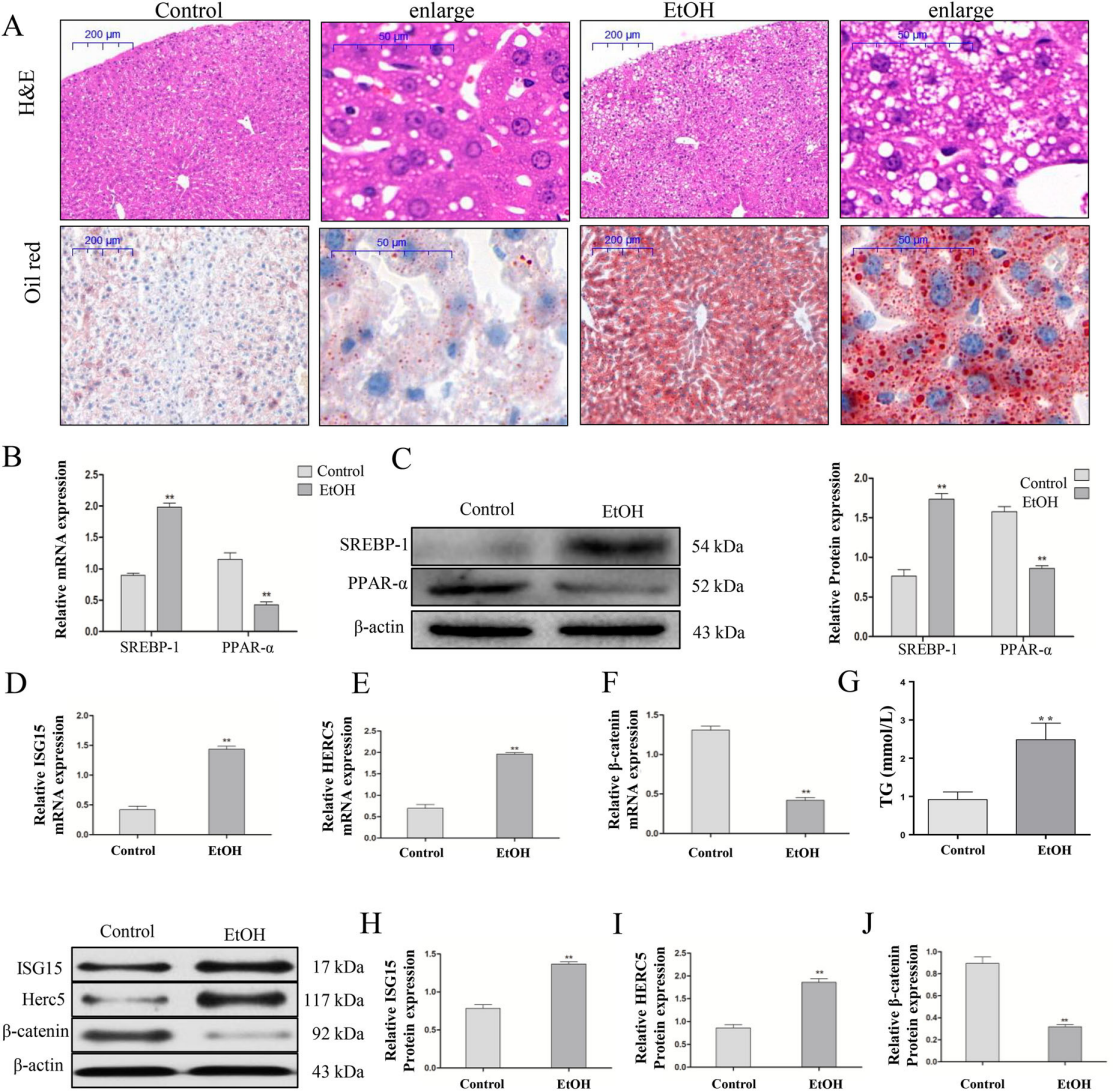
ISG15 and HERC5 levels were increased and β-catenin levels were decreased in the liver tissue of the EtOH-Fed Mice. (A) Establishment of an alcoholic fatty liver model. Hematoxylin and eosin (H&E) staining and Oil Red staining of alcoholic fatty liver mice liver sections (50×, 200×), Data represent the mean ± SD for 6–8 mice. (B-C) The expression levels of SREBP-1 and PPAR-α were detected by qRT-PCR and western blotting. (D-F) The mRNA expression levels of ISG15, HERC5, and β-Catenin were detected by qRT-PCR. (G) Results of the triglyceride (TG) levels of the hepatic tissue. (H-J) The protein expression levels of ISG15, HERC5, and β-catenin were detected by qRT-PCR and western blotting. *p < 0.05, **p < 0.01 versus control group. Data represent the mean ± SD for 3–4 independent experiments.

All male C57BL/6J mice with chronic alcohol feeding were characterized by inflammation, injury, and steatosis in the liver. H&E staining showed that the liver tissues in the EtOH-fed mice exhibited fat vacuoles, liver cell cord derangement, intercellular space dilatation, and inflammatory cell infiltration, whereas the liver tissues of the CD-fed mice showed normal lobular architecture with central veins and radiating hepatic cords. Oil red staining showed that the hepatic lipid deposition in the EtOH-fed group was significantly higher than in the CD-fed mice (Figure 1A). Additionally, qRT-PCR and western blotting showed that in the EtOH-fed group, SREBP-1 mRNA and protein levels were significantly increased, while PPAR-α mRNA and protein levels were significantly decreased compared with the control group (Figure 1B-C). These results suggest that the mouse model of AFLD was successfully established. Moreover, we found that the ISG15 and HERC5 mRNA and protein levels were significantly increased (Figure 1D,E,H,I) and that the mRNA and protein expression of β-catenin was significantly decreased (Figure 1F,I) in the liver tissues of the EtOH-fed group compared with normal liver tissues by qRT-PCR and western blotting, respectively. Moreover, the intrahepatic TG levels (Figure 1G) was also increased in the EtOH group compared with the control group.

### ISG15 and HERC5 were statistically increased and β-catenin was statistically decreased in the EtOH-stimulated AML-12 and L02 cells

**Figure 2.**
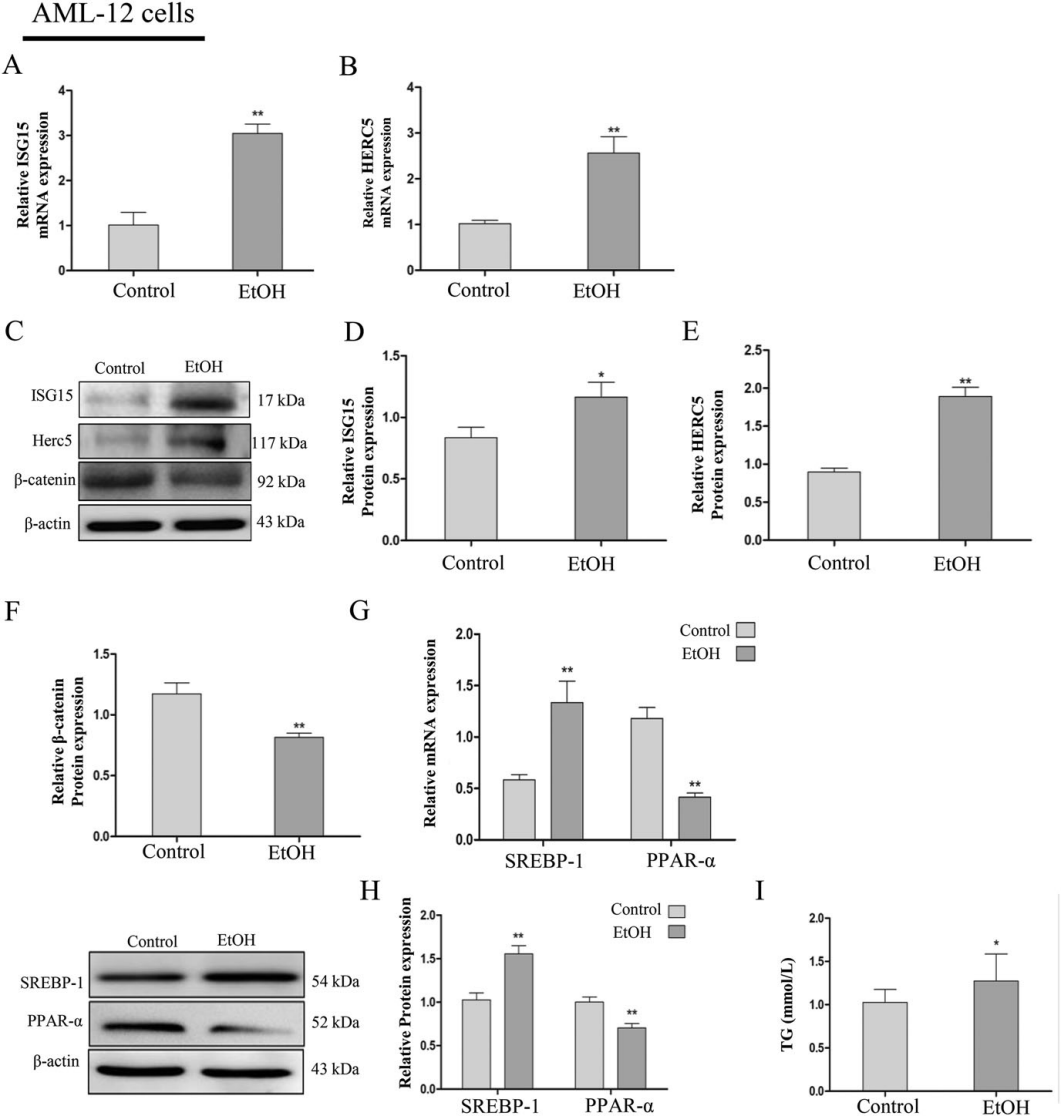
The model of EtOH-stimulated AML-12 cells wherein ISG15, and HERC5 had high expression levels and β-catenin had low expression levels. (A-B) The expression levels of ISG15 and HERC5 were detected by qRT-PCR. (C-E) The expression levels of ISG15 and HERC5 were detected by western blotting. (F-G) The expression levels of β-Catenin were detected by qRT-PCR and western blotting. (H) The expression levels of SREBP-1 and PPAR-α were detected by qRT-PCR. (I) The expression levels of SREBP-1, PPAR-α were detected by western blotting. (J) The expression of TG levels in cell supernatants. *p < 0.05, **p < 0.01 versus control group. Data represent the mean ± SD for 3–4 independent experiments.

**Figure 3.**
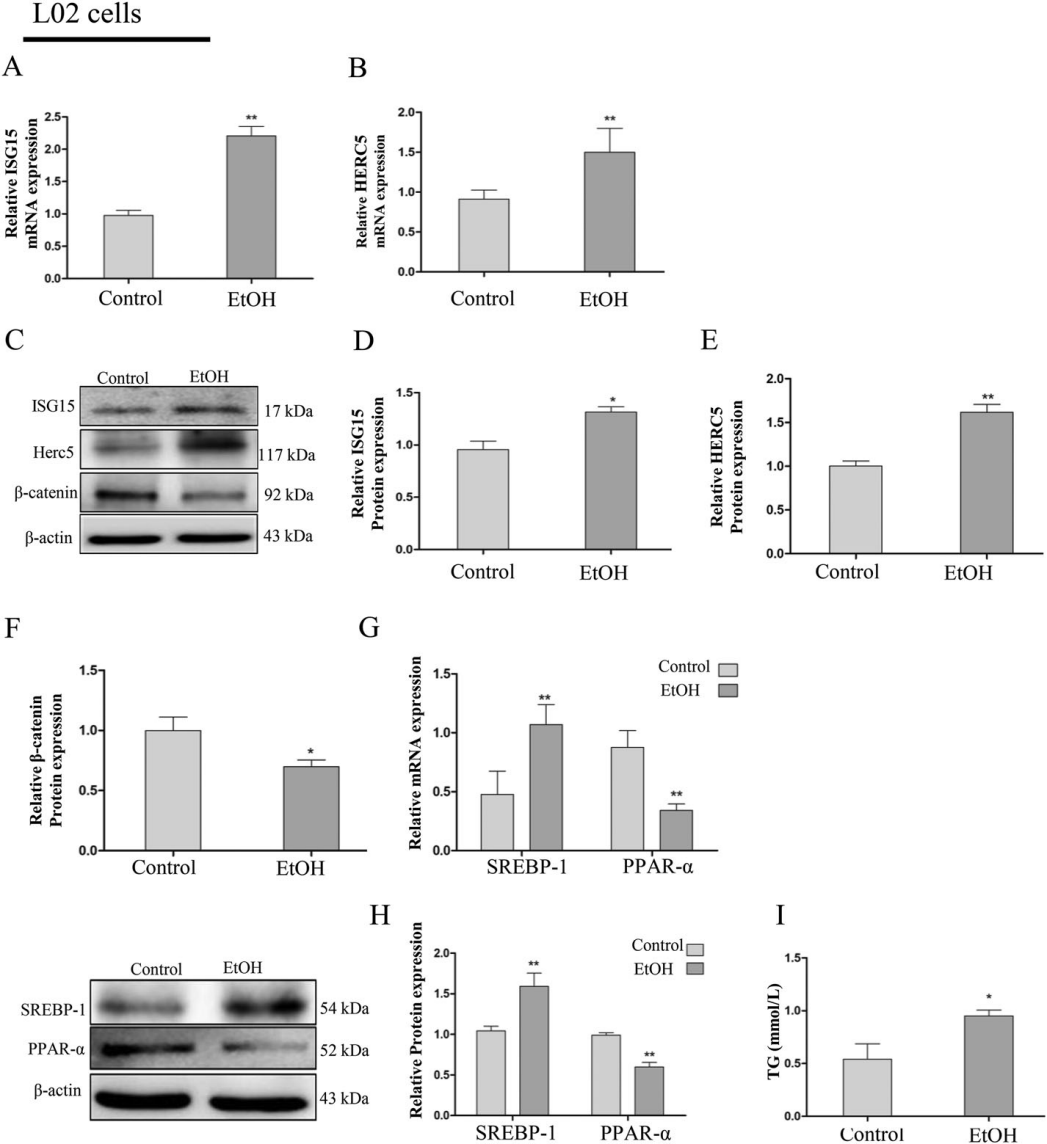
ISG15 and HERC5 levels were increased and β-catenin levels were decreased in EtOH-stimulated L02 cells. (A-B) The expression levels of ISG15 and HERC5 were detected by qRT-PCR. (C-E) The expression levels of ISG15 and HERC5 were detected by western blotting. (F-G) The expression levels of β-Catenin were detected by qRT-PCR and western blotting. (H) The expression levels of SREBP-1 and PPAR-α were detected by qRT-PCR. (I) The expression levels of SREBP-1 and PPAR-α were detected by western blotting. (J) The expression of TG levels in cell supernatants. *p < 0.05, **p < 0.01 versus control group. Data represent the mean ± SD for 3–4 independent experiments.

Then, we stimulated AML-12 cells with 100 mM EtOH and L02 cells with 150 mM EtOH for 24 h, wherein the concentrations were confirmed by the preliminary test. The results revealed that ISG15 and HERC5 mRNA and protein levels were elevated (Figure 2A-E) and β-catenin was reduced (Figure 2C, F) in AML-12 cells, which is consistent with findings on the previously described EtOH-fed mouse liver tissues. Likewise, in L02 cells, ISG15 and HERC5 expressions were elevated (Figure 3A-E) and β-catenin was decreased (Figure 3 C, F) at the protein level.

To determine the effect of EtOH ingestion on lipid homeostasis, the levels of SREBP-1 and PPAR-α were determined by qRT-PCR and western blotting. The results suggest that with the lower expression of PPAR-α, there was a significant increase in SREBP-1 expression in the AML-12 cells (Figure 2G-H). Furthermore, there was also an increase in TG levels in the cellular supernatant of the EtOH group compared with the control group (Figure 2I). The changes in levels of SREBP-1, PPAR-α (Figure 3G-H), and TG (Figure 3I) were consistent with the AML-12 cells and the previously described EtOH-fed mouse liver tissues. The above results indicate that the expression of ISG15 is positive and β-catenin is negative in AFLD and is closely related to lipid metabolism.

### β-catenin is stabilized in ISG B, C, and G sequences

**Figure 4.**
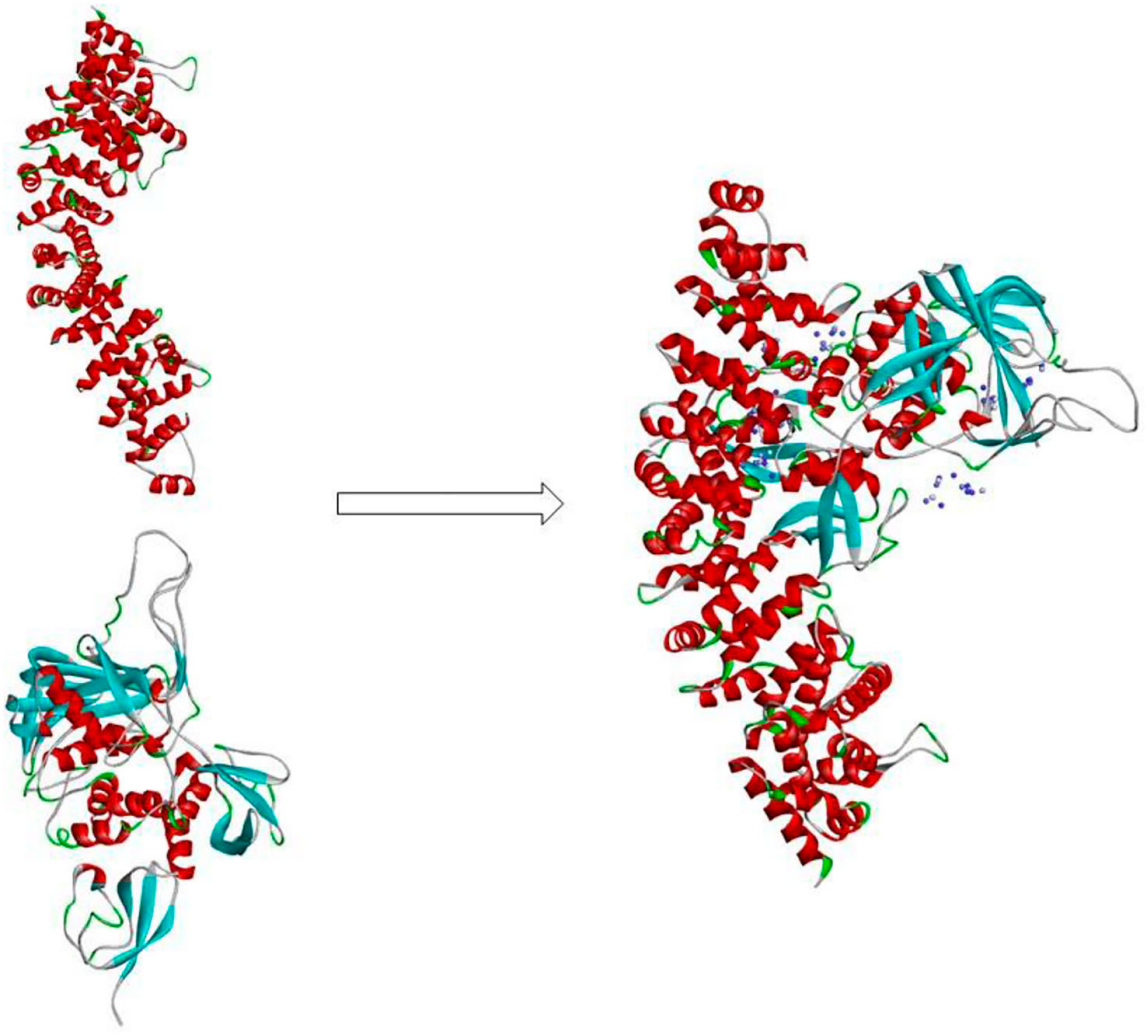
β-catenin tended to bind and stabilize the ISG15 B, C, and G sequences.

As other studies have reported, ISG15 binds to β-catenin through HERC5 (ISG15 E3 ligase) causing ISGylation, which leads to β-catenin degradation through the 20S proteasome pathway. To further study the connection between ISG15 and β-catenin, we used computer simulation docking technology to verify the interaction of ISG15 and β-catenin. The results showed that the protein molecules of the receptor ISG15 (PDB:6YVA) and the ligand β-catenin (PDB:4R0Z) form a stable docking structure (Figure 4) by salt bridge, hydrogen bond, π-π interaction. These results suggest that ISG15 can form a stable binding interaction with β-catenin, which may affect the expression of β-catenin.

### Downregulation of HERC5 using siRNA led to upregulation at the protein level of β-catenin, thereby alleviating lipidosis in EtOH-stimulated L02 cells

**Figure 5.**
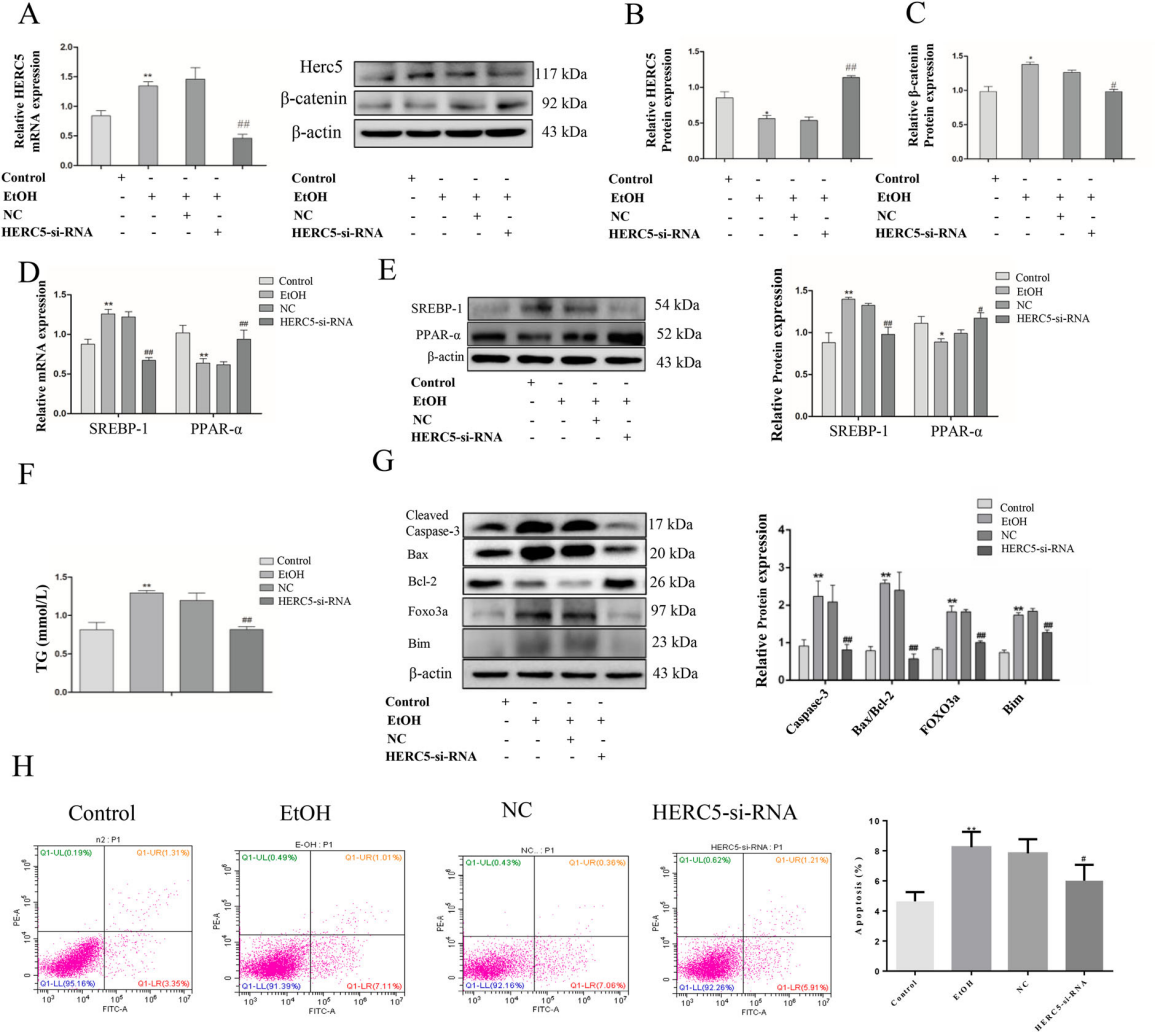
*HERC5*-siRNA downregulated the levels of β-catenin in EtOH-stimulated L02 cells. (A) Result of the qRT-PCR analysis of *HERC5* mRNA expression. (B-C) Results of western blotting analysis of HERC5 and β-Catenin. (D-E) *HERC5*-siRNA alleviated lipid metabolism disorders in EtOH-stimulated L02 cells. Results of the qRT-PCR and western blotting analysis of SREBP-1and PPAR-α. (F) The expression of TG levels in cell supernatants. (G) The apoptosis protein expression of FOXO3a, BIM, Cleaved-Caspase-3, Bax and Bcl-2 were analyzed by western blotting. (H) The levels of apoptosis were analyzed by flow cytometry. *p < 0.05, **p < 0.01 versus control group. *#p < 0.05 or ##p < 0.01* versus NC group. Data represent the mean ± SD for 3–4 independent experiments.

SiRNA that is specific for human *HERC5* was used to knockdown HERC5 expression (Figure 5A-B). After silencing the *HERC5* gene, the protein levels of β-catenin were detected by western blotting. These results showed that *HERC5*-siRNA significantly increased β-catenin protein expression (Figure 5C). The above results suggest that HERC5 downregulates the protein levels of β-catenin.

To affirm the role of *HERC5*-siRNA in lipid metabolism, the mRNA and protein expressions of SREBP-1 and PPAR-α were detected by qRT-PCR and western blotting, respectively. It was found that the SREBP-1 expression was decreased and the PPAR-α expression was elevated in the EtOH-stimulated L02 cells (Figure 5D-E). Furthermore, biochemical indicator test results showed that *HERC5*-siRNA significantly reduced TG levels in the cell supernatants (Figure 5F). These results demonstrate that *HERC5*-siRNA could alleviate lipid metabolism disorders.

### Downregulation of HERC5 by siRNA reduced apoptosis in EtOH-stimulated L02 cells

We also performed tests for apoptosis-related aspects wherein western blotting was used to detect the protein expression of the apoptotic genes *FOXO3* and *BIM*. *HERC5*-siRNA significantly reduced the protein expression of *FOXO3a* and its downstream target gene *BIM* (Figure 5G). Cleaved-Caspase-3 and the ratio of Bax/Bcl-2 protein levels were also markedly decreased in cells transfected with *HERC5*-siRNA (Figure 5G). Moreover, cell apoptosis was detected by flow cytometry, and our results showed that *HERC5*-siRNA significantly suppressed cell apoptosis (Figure 5H). These results indicated that silencing *HERC5* inhibits the apoptosis of L02 cells by inhibiting the *FOXO3* and *BIM genes*.

### Overexpressed HERC5 downregulated the expression of β-catenin protein and, aggravated lipidosis

**Figure 6.**
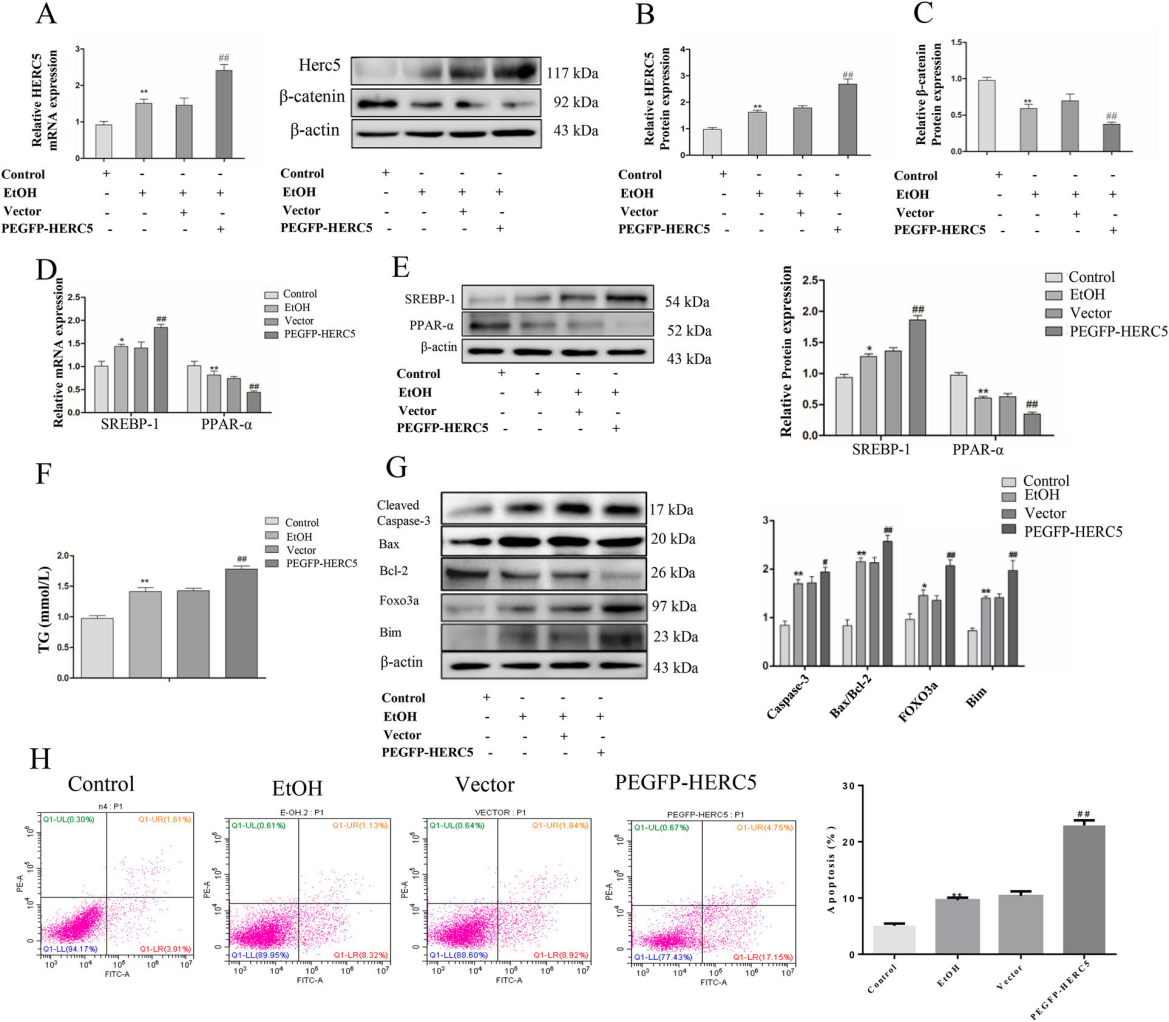
Overexpressed HERC5 upregulated the levels of β-catenin ISGylation in EtOH-stimulated L02 cells. (A) Results of the qRT-PCR analysis of *HERC5* mRNA expression. (B-C) Results of the western blotting analyses of HERC5 and β-Catenin. (D-E) Overexpressed HERC5 aggravated lipid metabolism disorders in EtOH-stimulated L02 cells. Results of the qRT-PCR and western blotting analyses of SREBP-1 and PPAR-α. (F) The expression of TG levels in cell supernatants. (G) The apoptosis protein expression of FOXO3a, BIM, Cleaved-Caspase-3, Bax and Bcl-2 were analyzed by western blotting. (H) The levels of apoptosis were analyzed by flow cytometry. *p < 0.05, **p < 0.01 versus control group. *#p < 0.05 or ##p < 0.01* versus Vector group. Data represent the mean ± SD for 3–4 independent experiments.

Conversely, we used pEGFP-*HERC5* vector to construct a model exhibiting an overexpression of HERC5 (Figure 6A-B). We then detected β-catenin using the same previously described methods. The data showed that the protein levels of β-catenin were significantly decreased (Figure 6C). The above results showed that high expression of HERC5 downregulated the levels of β-catenin in EtOH-stimulated L02 cells.

We detected the levels of SREBP-1 and PPAR-α in the EtOH-stimulated L02 cells by qRT-PCR and western blotting as previously described. The results showed that SREBP-1 was upregulated and PPAR-α was downregulated both at the mRNA and protein levels (Figure 6D-E). Consistent with previous findings, our data indicated that TG levels were significantly increased in the cell supernatant (Figure 6F). These results suggest that high expression of HERC5 aggravates lipid metabolism disorders.

### Overexpressed HERC5 increased apoptosis in EtOH-stimulated L02 cells

Western blotting results showed that the apoptotic genes *FOXO3* and *BIM* were significantly increased (Figure 6G). Overexpressed HERC5 significantly induced the protein expression of Cleaved-Caspase-3 and the ratio of Bax/Bcl-2 (Figure 6G). Flow cytometry results showed that pEGFP-*HERC5* markedly increased cell apoptosis (Figure 6H). The above results indicate that a high expression of HERC5 increases the apoptosis of L02 cells by upregulating the *FOXO3* and *BIM* genes.

### The mechanism of β-catenin on lipid metabolism and apoptosis in EtOH-stimulated L02 cells

**Figure 7.**
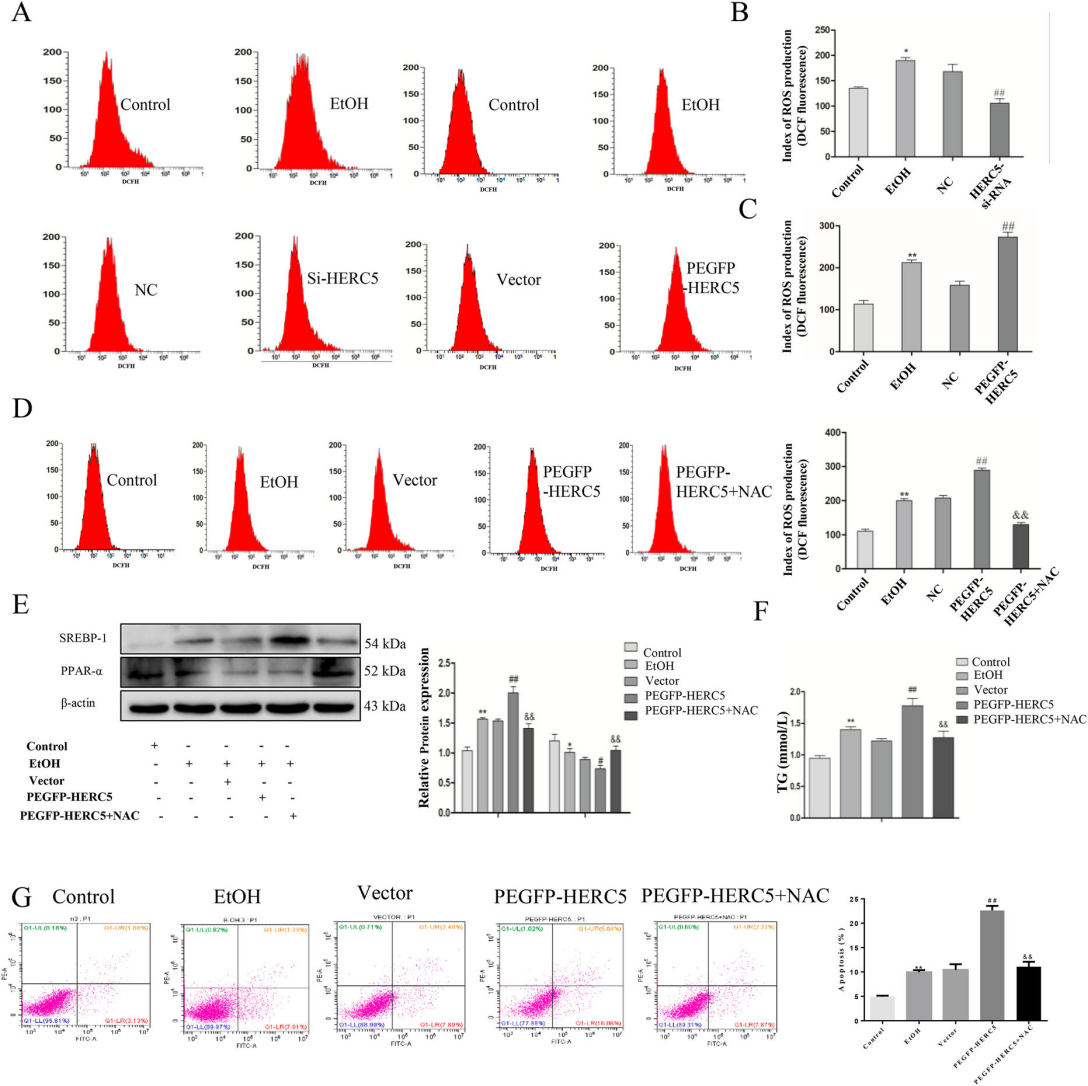
The ISGylation of β-catenin can regulate cell lipid metabolism by altering ROS levels. (A-C) Results of ROS levels in L-02 cells with *HERC5*-siRNA and pEGFP-*HERC5* transfection. (D) Results of the ROS levels in L-02 cells with addition of NAC. (E) Results of the western blotting analysis of SREBP-1 and PPAR-α. (F) Results of the TG analysis. β-catenin ISGylation affects cell apoptosis by regulating ROS levels. (G) The levels of apoptosis were detected by flow cytometry. *p < 0.05, **p < 0.01 versus control group. *#p < 0.05, ##p < 0.01* versus NC/Vector group. *&p < 0.05, &&p < 0.01* versus pEGFP-*HERC5*. Data represent the mean ± SD for 3–4 independent experiments.

Levels of ROS in the EtOH-stimulated L02 cells were detected by flow cytometry, and the results indicated that low expression of HERC5 was associated with significantly decreased levels of ROS (Figure 7A-B). Next, this same test for ROS was performed on the EtOH-stimulated L02 cells transfected with the plasmid pEGFP-*HERC5*. The results showed that the ROS levels increased significantly (Figure 7A, C). This experiment was repeated with the addition of the ROS inhibitor, N-acetylcysteine (NAC), to the EtOH-stimulated L02 cells after transfection with the plasmid pEGFP-*HERC5*. The flow cytometry results showed that with the addition of NAC, ROS levels decreased significantly (Figure 7D).

To further explore whether β-catenin affects lipid metabolism and cell apoptosis by regulating ROS, the following experiment was performed. Levels of SREBP-1 were significantly reduced, while levels of PPAR-α were significantly elevated (Figure 7E). The results showed that the TG levels were significantly decreased (Figure 7F). The studies on apoptosis indicated that after the addition of NAC to cells, apoptosis was significantly reduced (Figure 7G). These results indicate that NAC can reverse excessive lipid deposition and apoptosis caused by overexpression of HERC5.

## Discussion

Alcohol has been classified as a carcinogen by the International Agency for Cancer Research (IACR) of WHO [17], and continuing alcohol ingestion is the most important risk factor for liver cancer progression. The initial stage of liver injury caused by persistent alcohol stimulation involves lipid degeneration and fat accumulation, which strongly influence lipid metabolism. Increased lipogenesis and excessive lipid metabolism disorders directly damage hepatocytes, leading to an inflammatory response, oxidative stress, and cytokine production, resulting in the development of AFLD [18]. Therefore, preventing the development of hepatic lipid metabolism disorders in patients exposed to persistent alcohol can prevent further liver damage.

Studies have shown that blocking the Wnt/β-catenin pathway leads to the development of oxidative defects of fatty acids, increased oxidative stress, decreased expression of ethanol metabolic enzymes, the development of liver gluconeogenesis defects, etc [19]. High expression of β-catenin can alleviate chronic liver injury induced by alcohol [20–22]. Furthermore, the protein stability of β-catenin affects its expression. Therefore, the regulation of the protein stability of β-catenin is significant to the pathogenesis of ALD. Post-translational modifications such as ubiquitination, phosphorylation, SUMOylation, and acetylation play an important role in regulating protein stability. Recent studies have reported that ISG15 can bind to β-catenin through the E3 ligase HERC5, causing ISG degradation and affecting the protein expression of β-catenin [12].

In this study, we found that HERC5 and ISG15 levels were significantly higher and β-catenin levels were significantly lower in the liver tissue from the EtOH-fed mice than in the liver tissue from the CD-fed mice. To further illustrate the potential function of HERC5, ISG15, and β-catenin in the progression of ALD, we induced liver injury in the AML-12 and L02 cells with EtOH in vitro. The results showed that HERC5 and ISG15 levels were significantly upregulated and β-catenin levels were significantly downregulated after the EtOH stimulation. Moreover, we studied a computer simulation using docking technology to confirm that β-catenin forms a stable complex structure with ISG15. This indirectly proves that the protein stability of β-catenin affects its expression. It has been reported that β-catenin can be modified by ISG15. Furthermore, HERC5, an E3 ligase, was found to be necessary for the ISGylation of β-catenin. This was observed in this study when *HERC5* was silenced using siRNA, causing an upregulation of β-catenin levels. Moreover, the overexpression of HERC5 with pEGFP-*HERC5* caused a downregulation of β-catenin levels. These findings further confirm that the protein levels of β-catenin are downregulated by HERC5. SREBP-1, an important transcription factor that accelerates fatty acid synthesis, has been implicated in the pathogenesis of AFLD [23,24]. Similarly, PPAR-α is a nuclear hormone receptor that can modulate the transport and oxidation of fatty acids [25]. The above proteins regulate lipogenesis and β-oxidation in hepatocytes [26]. Our studies have indicated that SREBP-1 levels were upregulated while PPAR-α levels were downregulated in EtOH-fed mice and EtOH-stimulated AML-12 and L02 cells. It was observed that lower expression of HERC5 suppressed the ethanol-mediated decrease in the levels of SREBP-1 and TG but increased the ethanol-mediated elevation in the levels of PPAR-α. On the contrary, when HERC5 was overexpressed, the opposite result was achieved. It was also observed that lower expression of HERC5 was correlated with lower levels of apoptosis and vice versa. Therefore, all these findings suggest that β-catenin ISGylation in AFLD is closely related to the lipid metabolism and apoptosis of liver cells.

Alcohol exposure increases ROS production and reduces antioxidant levels in cells, leading to hepatocyte apoptosis and hepatic inflammation [3]. Accumulating evidence suggests that the inhibition of oxidative stress and apoptotic hepatocytes are critical approaches to alleviate ethanol-induced liver injury [27,28]. In our study, ROS levels were significantly increased after ethanol stimulation. Aberrant Wnt/β-catenin signaling altered the production of ROS in the EtOH-stimulated cells. We also regulated HERC5 with *HERC5*-siRNA or pEGFP-*HERC5*. The results showed that *HERC5*-siRNA reduced the levels of ROS while pEGFP-*HERC5* increased the levels of ROS. Moreover, inhibition of ROS production with NAC can reverse excessive lipid metabolism disorders and apoptosis. Thus, HERC5-mediated ISGylation decreased β-catenin expression and enhanced ROS production.

## Conclusions

Taken together, we found that the expression of β-catenin is closely related to the lipid metabolism, apoptosis, and levels of ROS in AFLD. Our study contributed to the understanding of the regulatory mechanism of the Wnt/β-catenin signaling pathway in AFLD, preliminarily revealed the effect and possible mechanism of β-catenin ISGylation on AFLD, and provided a new therapeutic strategy for the clinical prevention and treatment of ALD.

## Data Availability

The authors confirm that the datasets used and analyzed during the current study are available from the corresponding author on reasonable request.
